# Genotypic and PFGE/MLVA Analyses of *Vibrio cholerae* O1: Geographical Spread and Temporal Changes during the 2007–2010 Cholera Outbreaks in Thailand

**DOI:** 10.1371/journal.pone.0030863

**Published:** 2012-01-24

**Authors:** Kazuhisa Okada, Amonrattana Roobthaisong, Ichiro Nakagawa, Shigeyuki Hamada, Siriporn Chantaroj

**Affiliations:** 1 Thailand-Japan Research Collaboration Center on Emerging and Re-emerging Infections, Nonthaburi, Thailand; 2 Research Collaboration Center on Emerging and Re-emerging Infections, Research Institute for Microbial Diseases, Osaka University, Osaka, Japan; 3 Section of Bacterial Pathogenesis, Tokyo Medical and Dental University Graduate School of Medical and Dental Sciences, Tokyo, Japan; 4 National Institute of Health, Department of Medical Sciences, Ministry of Public Health, Nonthaburi, Thailand; St. Petersburg Pasteur Institute, Russian Federation

## Abstract

**Background:**

*Vibrio cholerae* O1 El Tor dominated the seventh cholera pandemic which occurred in the 1960s. For two decades, variants of *V. cholerae* O1 El Tor that produce classical cholera toxin have emerged and spread globally, replacing the prototypic El Tor biotype. This study aims to characterize *V. cholerae* O1 isolates from outbreaks in Thailand with special reference to genotypic variations over time.

**Methods/Findings:**

A total of 343 isolates of *V. cholerae* O1 from cholera outbreaks from 2007 to 2010 were investigated, and 99.4% were found to carry the classical cholera toxin B subunit (*ctxB*) and El Tor *rstR* genes. Pulsed-field gel electrophoresis (PFGE) differentiated the isolates into 10 distinct pulsotypes, clustered into two major groups, A and B, with an overall similarity of 88%. Ribotyping, multiple-locus variable-number tandem-repeat analysis (MLVA), and PCR to detect *Vibrio* seventh pandemic island II (VSP-II) related genes of randomly selected isolates from each pulsotype corresponded to the results obtained by PFGE. Epidemiological investigations revealed that MLVA type 2 was strongly associated with a cholera outbreak in northeastern Thailand in 2007, while MLVA type 7 dominated the outbreaks of the southern Gulf areas in 2009 and MLVA type 4 dominated the outbreaks of the central Gulf areas during 2009–2010. Only MLVA type 16 isolates were found in a Thai-Myanmar border area in 2010, whereas those of MLVA types 26, 39, and 41 predominated this border area in 2008. Type 39 then disappeared 1–2 years later as MLVA type 41 became prevalent. Type 41 was also found to infect an outbreak area.

**Conclusions:**

MLVA provided a high-throughput genetic typing tool for understanding the in-depth epidemiology of cholera outbreaks. Our epidemiological surveys suggest that some clones of *V. cholerae* O1 with similar but distinctive genetic traits circulate in outbreak sites, while others disappear over time.

## Introduction

The bacterium *Vibrio cholerae* causes cholera, an acute infectious diarrheal disease that can result in death without appropriate treatment. More than 200 serogroups of *V. cholerae* are known to date, but only serogroups O1 and O139 are know to cause cholera of epidemic and pandemic proportions [1]. The O1 serogroup is divided into three serotypes, Ogawa, Inaba, and Hikojima, and two biotypes, classical and El Tor. Classical biotype strains have been responsible for the sixth cholera pandemic which spanned from 1899 to 1923, while El Tor biotype strains caused the seventh cholera pandemic, which began to spread worldwide in 1961 [2].

Since the early 1990s, new variants of *V. cholerae* O1 El Tor that possess traits of both classical and El Tor biotypes have emerged [3]. Several studies reported that El Tor variants have replaced prototypic El Tor strains in several Asian and African countries [4]–[14]. For example, in Bangladesh, all El Tor isolates of *V. cholerae* O1 obtained since 2001 have produced classical cholera toxin [6]. In Kolkata, India, El Tor variant strains carrying the El Tor *rstR* gene (CTX prophage repressor gene) and the classical cholera toxin B subunit (*ctxB*) gene, have superseded the El Tor type *ctxB* since 1995 [4], [5], while in northern Vietnam, El Tor variants carrying the El Tor *rstR* and the classical *ctxB* genes have been reported since late 2007 [11]. Recently, the World Health Organization reported that *V. cholerae* El Tor variant strains cause more severe episodes of cholera with higher fatality rates, compared with prototypic El Tor strains [15]. Due to these aspects of clinical manifestation and altered characteristics of cholera agents in recent years, more detailed investigations of cholera are required.

Several molecular typing tools have been used to depict genetic relatedness among *V. cholerae* isolates obtained from outbreak sites. In general, molecular markers of low variability can be used to establish phylogenetic relationships among isolates that have evolved over longer time spans, and highly variable markers discriminate closely related organisms for the surveillance of causative agents in cholera outbreaks. Ribotyping has been successfully used to typify *V. cholerae* O1 isolates from various countries [16] and is an appropriate tool for establishing phylogenetic relationships among organisms that have evolved over a longer time span. Pulsed-field gel electrophoresis (PFGE) has also been used to characterize clonal diversity and relationships among *V. cholerae* isolates. Although this is a powerful method for the routine subtyping of *V. cholerae* in detecting clusters of infection [17], it is not discriminatory enough to distinguish some epidemiologically unrelated *V. cholerae* O1 isolates [18], [19].

Multilocus variable-number tandem repeat (VNTR) analysis (MLVA) has been developed for a variety of bacterial pathogens [20]. This method is based on the variation in the number of repeats at multiple VNTR loci, which is highly variable. Danin-Poleg *et al.* [21] first reported the usefulness of MLVA to distinguish isolates of *V. cholerae*. The initial analysis of five VNTR loci revealed distinct populations in Bangladesh and India [22]–[24]. MLVA is a sophisticated method that can be useful for differentiating *V. cholerae* strains that would be indistinguishable by other techniques [21]. However, the potential value of MLVA as an epidemiological tool in the analysis of *V. cholerae* remains to be assessed.

In the present study, we characterized various *V. cholerae* O1 isolates collected from cholera outbreaks in Thailand between 2007 and 2010 using PFGE, ribotyping, MLVA and other tools, and investigated the origin(s) and appearance/disappearance of *V. cholerae* O1 El Tor variants over time.

## Methods

### Bacterial isolation

A total of 343 *V. cholerae* serogroup O1 isolates (Table S1) from cholera patients, their family members, and neighbors (n = 328), as well as environmental samples (n = 15) were investigated. *V. cholerae* strains 569B (classical biotype), N16961 (El Tor biotype), J16173 (Kolkata, India, 2004), and CE87 (Kolkata, India, 2004) were kindly supplied by Gopinath B. Nair, National Institute of Cholera and Enteric Diseases (NICED), Kolkata, India, and were used in this study for reference.

Human and environmental samples were cultured on Thiosulfate Citrate Bile Salts Sucrose Agar (Eiken, Tokyo, Japan). After overnight incubation, suspected *V. cholerae* colonies were confirmed by slide agglutination test with specific monoclonal antibodies (Denka Seiken, Tokyo, Japan) to identify the serogroup, O1 or O139, and their serotype, Ogawa or Inaba. A single colony was picked for each sample. Individual colonies were selected and cultured for 18 h at 37°C on Tryptic Soy Broth (TSB) (Difco, Detroit, MI). In addition, Loop-mediated isothermal amplification (LAMP) [25] was performed to screen for toxigenic *V. cholerae* among approximately 2,000 samples. Biotyping of the isolates was carried out using the Voges-Proskauer (VP) reaction and polymyxin B (50 units) [26]. Approval by ethical committee and patient consent were not obtained as this was considered a standard evaluation of an existing method, which has been undertaken as part of normal public health practice by the members of local public health authority. Furthermore the samples that we used were unlinked and anonymised so as to permanently protect patient confidentiality.

### DNA preparation

DNA templates for PCR and MLVA were extracted with the NucleoSpin Tissue Kit (Macherey-Nagel, Düren, Germany) and quantified using the NanoDrop ND-1000 spectrophotometer (Thermo Scientific, Illkirch, France). DNA samples were diluted to a concentration of 10 ng/µl and used as templates for gene detection and MLVA. For ribotyping, bacteria cultured in TSB for 18 h at 37°C were collected by centrifugation and suspended in TE buffer (10 mM Tris-HCl, 1 mM EDTA [pH 8.0]), treated with 10% (wt/vol) sodium dodecyl sulfate (SDS) and proteinase K (New England Biolabs, Beverly, MA), and incubated at 37°C for 1 h. After incubation, 10% Cetyltrimethylammonium bromide in 0.7 M NaCl was added and incubated at 65°C for 10 min [27]. The aqueous phase was treated with phenol-chloroform, and the DNA pellet was washed with 70% ethanol by centrifugation and resuspended in TE buffer, pH 8.0.

### PCR

PCR was carried out for detection of the *rtxC* [28] and *rstR* genes [29] encoding the acyltransferase and the CTX phage transcriptional regulator, respectively. Hexaplex PCR, which detects the presence of the virulence and regulatory genes *ctxA*, *zot*, *ace*, *tcpA*, *ompU* and *toxR*, was carried out to screen for toxigenic/pathogenic *V. cholerae* from both human and environmental samples [30]. The mismatch amplification mutation assay (MAMA) was used to detect sequence polymorphisms in CT genotype 1 (classical type CT) and genotype 3 (El Tor type CT) based on the nucleotide position 203 of the *ctxB* gene [31]. The presence or absence of ORFs in the *Vibrio* seventh pandemic island I (VSP-I) and *Vibrio* seventh pandemic island II (VSP-II) clusters of selected isolates was examined by PCR using primers described previously [32]–[34] (Table S2).

### Ribotyping

Genomic DNA from *V. cholerae* O1 isolates (2 µg) was digested with *Bgl*I (New England Biolabs), and the fragments were separated by electrophoresis in a 0.8% agarose gel and transferred to ultra-pure nylon membranes (Roche Diagnostic GmbH, Mannheim, Germany). *E. coli* ribosomal RNA specimens (16S and 23S) (Roche Diagnostic) were reverse transcribed into cDNA with SuperScript II reverse transcriptase (Invitrogen, Carlsbad, CA) [16] and labeled by random priming with digoxigenin-dUTP. Hybridization was performed with probes according to the manufacturer's instructions (Roche Diagnostic). The membranes were then visualized by the addition of alkaline phosphate-conjugated anti-digoxigenin antibody, and substrate disodium 3-(4-methoxyspiro{1,2-dioxetane-3,2′-[5′-chloro]tricyclo[3.3.1.1^3,7^] decan}-4-yl] phenyl phosphate. The membrane was developed, and results were recorded on x-ray film.

### PFGE

PFGE analysis was performed according to a PulseNet standardized protocol for *V. cholerae* subtyping [17] with minor modifications. Briefly, organisms were transferred into Tris-EDTA (100 mM each) buffer (pH 8.0) and adjusted to an OD of 0.8–1.0 at 610 nm. Agarose plugs were prepared by mixing equal volumes of the adjusted bacterial suspension with 200 µl of melted 1.0% Pulse Field Certified agarose (Bio-Rad) and 10 µl of proteinase K (20 mg/ml stock) (New England Biolabs). Organisms in the agarose plugs were lysed in a lysis solution (Tris-EDTA (50 mM each; pH 8.0), 1% sarcosine, 0.5 mg/ml proteinase K) for 1 h at 54°C. Washing was performed in six stages, twice with sterile water and four times with Tris-EDTA buffer. One section of the plug was equilibrated with NEBuffer 3 (New England Biolabs), placed in 200 µl fresh buffers containing 40 U *Not*I and incubated for at least 4 h at 37°C. Standard Lambda ladder (Bio-Rad) was used as a DNA molecular mass marker. The digested chromosomal DNA was subjected to PFGE on a 1% Pulse Field Certified agarose in 0.5× Tris-borate-EDTA at 14°C using CHEF-DRIII system (Bio-Rad). The pulse time ranged from 2–10 s for 13 h and from 20–25 s for 6 h at 6 V. PFGE banding patterns were analyzed with computer software BioNumerics version 6.1 (Applied Maths, Kortrijk, Belgium), and a dendrogram was produced using the Dice coefficient and the unweighted pair-group method with arithmetic mean algorithm (UPGMA) with a position tolerance of 1.3%.

### MLVA/VNTR

We selected five VNTR loci that exhibited high diversity indexes as reported previously [21]. These loci were amplified using specific primers [22] and could be identified by those genes in the order of occurrence: VC0147, VC0436-7, VC1650, VC0171, and VCA0283. PCR products were purified using the NucleoSpin Extract II Kit (Macherey-Nagel), sequenced using the BigDye Terminator v3.1 Cycle Sequencing Kit (Applied Biosystems, Foster City, CA) and loaded onto an ABI 3130*xl* automated sequencer (Applied Biosystems) according to the manufacturer's instructions. Sequence data for each isolate were read using BioEdit Sequence Alignment Editor v.7.0.5.3 (http://www.mbio.ncsu.edu/bioedit/bioedit.html) [35]. The numbers of repeats in an alignment were counted and listed sequentially for the five VNTR loci to generate an isolate pattern. For example, the pattern 10, 6, 7, 18, 18 indicates ten repeats at locus VC0147, six at locus VC0436-7, and so on. The resulting data were imported into BioNumerics software version 6.1. Cluster analysis was performed using the categorical and the UPGMA options. The diversity of PFGE and MLVA types was assessed using Simpson's diversity index [36]. Confidence intervals (CI) were calculated as previously described [37].

## Results

### Serotypes, biotypes, and types of virulence genes of *Vibrio cholerae* O1 isolates

All *V. cholerae* O1 isolates used in this study were found to be serogroup O1; 166 of 343 isolates belonged to serotype Ogawa, while the remainder were serotype Inaba (Table 1). Polymyxin B sensitivity, VP testing and PCR analysis for the *rtxC* gene indicated that all isolates were biotype El Tor, and the hexaplex PCR assay revealed that they contained a set of virulence genes and were positive for the El Tor-specific *tcpA* gene; however, one isolate did not carry *rstR*, *ctxA*, *ctxB*, *ace*, and *zot* genes. MAMA-PCR showed that 341 isolates harbored the classical *ctxB* genotype and one isolate carried the El Tor *ctxB* genotype (Table 1). PCR for the allele-specific CTX prophage repressor gene (*rstR*) revealed that the isolates produced amplicons of El Tor *rstR* only.

**Table 1:** Characterization of *V. cholerae* O1 isolates used in this study.

Province	Year of isolation	Source	No. of isolates	Serotype	*ctxB*	*rstR*	*rtxC*	*ace*	*zot*	*ctxA*	*tcpA*	*toxR*	*ompU*	*Type*
Lamphun*	2007	Human	1	Ogawa	Cl†	El	+‡	+	+	+	El	+	+	(i)
Khon Kaen*	2007	Human	6	Ogawa	Cl	El	+	+	+	+	El	+	+	(i)
Udonthani*	2007	Human	4	Ogawa	Cl	El	+	+	+	+	El	+	+	(i)
Samutsakorn	2008	Human	1	Ogawa	Cl	El	+	+	+	+	El	+	+	(i)
Tak	2008	Human	1	Ogawa	Cl	El	+	+	+	+	El	+	+	(i)
Prachuapkirikhan	2009	Human	2	Ogawa	Cl	El	+	+	+	+	El	+	+	(i)
Samutsakorn	2009	Human	5	Ogawa	Cl	El	+	+	+	+	El	+	+	(i)
Tak	2009	Human	6	Ogawa	Cl	El	+	+	+	+	El	+	+	(i)
Songkla	2009	Human	7	Ogawa	Cl	El	+	+	+	+	El	+	+	(i)
Pattani	2009	Human	25	Ogawa	Cl	El	+	+	+	+	El	+	+	(i)
Patthalung	2009	Human	4	Ogawa	Cl	El	+	+	+	+	El	+	+	(i)
Narathiwat	2009	Human	2	Ogawa	Cl	El	+	+	+	+	El	+	+	(i)
Samutsakorn	2010	Human	11	Ogawa	Cl	El	+	+	+	+	El	+	+	(i)
Tak	2010	Human	83	Ogawa	Cl	El	+	+	+	+	El	+	+	(i)
Tak	2010	Env	3	Ogawa	Cl	El	+	+	+	+	El	+	+	(i)
Tak (import)	2010	Human	3	Ogawa	Cl	El	+	+	+	+	El	+	+	(i)
Rachaburi	2008	Human	1	Inaba	Cl	El	+	+	+	+	El	+	+	(ii)
Tak	2008	Human	98	Inaba	Cl	El	+	+	+	+	El	+	+	(ii)
Tak	2008	Env	8	Inaba	Cl	El	+	+	+	+	El	+	+	(ii)
Tak	2009	Human	29	Inaba	Cl	El	+	+	+	+	El	+	+	(ii)
Tak	2009	Env	2	Inaba	Cl	El	+	+	+	+	El	+	+	(ii)
Samutsakorn	2010	Human	8	Inaba	Cl	El	+	+	+	+	El	+	+	(ii)
Samutsakorn	2010	Env	2	Inaba	Cl	El	+	+	+	+	El	+	+	(ii)
Narathiwat	2010	Human	1	Inaba	Cl	El	+	+	+	+	El	+	+	(ii)
Tak	2010	Human	28	Inaba	Cl	El	+	+	+	+	El	+	+	(ii)
Tak	2008	Human	1	Ogawa	El	El	+	+	+	+	El	+	+	(iii)
Tak	2010	Human	1	Ogawa	−	−	+	−	−	−	El	+	+	(iv)
Reference strains														
India, J16173	2004			Inaba	Cl	El	+	+	+	+	El	+	+	
India, CE87	2004			Inaba	Cl	El	+	+	+	+	El	+	+	
N16961 (El Tor)	1971			Inaba	El	El	+	+	+	+	El	+	+	
569B (Classical)	1948			Inaba	Cl	Cl	−	+	+	+	Cl	+	+	

*Data from Okada *et al* [13].

† Cl, Classical allele; El, El Tor allele.

‡ +, Positive; −, Negative.

Thus, two major types were found among the 343 *V. cholerae* O1 isolates: *V. cholerae* O1 El Tor carrying the classical *ctxB* and the El tor *rstR* genes and of serotype Ogawa (47.8%, type i), or serotype Inaba (51.6%, type ii). In addition, two minor types were found: *V. cholerae* O1 El Tor, serotype Ogawa, carrying the *ctxB* and *rstR* genes of the El Tor type (0.3%, type iii), and non-toxigenic *V. cholerae* O1 El Tor, serotype Ogawa lacking the CTX elements (0.3%, type iv) (Table 1).

### Variation, prevalence and relatedness of PFGE pulsotypes and MLVA types among *V. cholerae* O1 isolates

**Figure 1:**
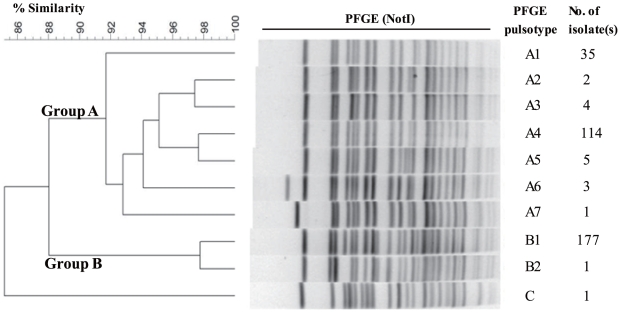
Pulsed-field gel electrophoresis (PFGE) patterns among 343 Thai *V. cholerae* O1 isolates. PFGE banding patterns were analyzed with computer software, BioNumerics version 6.1 and a dendrogram was produced using the Dice coefficient and UPGMA algorithm. *V. cholerae* O1 isolate pulsotypes of the El Tor variant were categorized into groups A and B, with an overall similarity of 88%.

PFGE of *Not*I digests of the 343 isolates differentiated into 10 pulsotypes (Figure 1). UPGMA clustering grouped the PFGE patterns into two major groups, A and B, with 88% overall relatedness. Group A comprised 7 pulsotypes (A1–A7) which exhibited 92% similarity, while group B presented 2 pulsotypes (B1 and B2), showing 98% similarity. Pulsotypes A1 and A4 were prevalent among the isolates of serotype Ogawa, whereas pulsotype B1 was predominantly found in serotype Inaba. The other 7 pulsotypes (5.0% of all isolates) were also classified. The *V. cholerae* O1 El Tor serotype Ogawa carrying the El Tor *ctxB* gene (type iii, Table 1) was excluded from the two groups (Figure 1). One isolate of non-toxigenic *V. cholerae* O1 El Tor (type iv) belonged to pulsotype A5.

Arbitrarily selected isolates from each pulsotype were subjected to ribotyping and PCR. The PFGE group A isolates and strain J16173 exhibited ribotype RIII, while group B isolates and strain CE87 were classified as ribotype RIV (Table 2). PCR revealed that group A isolates and strain J16173 carried the VC0502 gene, which encodes a type IV pilin, on the VSP-II, whereas the ORF was absent in group B isolates and strain CE87 (Table 2). The isolates of both clusters lacked spanning genes VC0495–VC0496 on the VSP-II, but commonly possessed gene VC0514.

**Table 2:** Ribotyping and virulence-related gene analyses of arbitrarily selected *V. cholerae* O1 isolates from each PFGE pulsotype.

					*Vibrio* seventh pandemic island cluster			
					VSPI	VSPII		
PFGE	Sample ID no.	Serotype	Ribotype	*ctxB* sequence	VC0174-0186	VC0502	VC0514	VC0495-0496
**A1**	KK24	Ogawa	RIII	Classical	∼15 kb	+†	+	−
**A2**	MS83	Ogawa	RIII	Classical	∼15 kb	+	+	−
**A3**	MS84A	Ogawa	RIII	Classical	∼15 kb	+	+	−
**A4**	MRM1A	Ogawa	RIII	Classical	∼15 kb	+	+	−
**A5**	TSY419	Ogawa	RIII	Classical	∼15 kb	+	+	−
**A6**	TSY216	Ogawa	RIII	Classical	∼15 kb	+	+	−
**A7**	TSY373	Ogawa	RIII	Classical	∼15 kb	+	+	−
**B1**	PP34	Inaba	RIV	Classical	∼15 kb	−	+	−
**B2**	R4051-225511	Inaba	RIV	Classical	∼15 kb	−	+	−
**C**	MS6	Ogawa	Unknown	El Tor	ND*	ND	ND	ND
**Reference strains**								
**H** ‡	J16173	Inaba	RIII‡	Classical	∼15 kb	+	+	−
**H1** ‡	CE87	Inaba	RIV‡	Classical	∼15 kb	−	+	−
**ND**	N16961	Inaba	ND	El Tor	∼15 kb	+	+	+
**ND**	569B	Inaba	ND	Classical	1.3 kb	−	−	−

*Not Determined.

† +, positive; −, negative.

‡ Data from Raychoudhuri *et al* [40].

**Figure 2:**
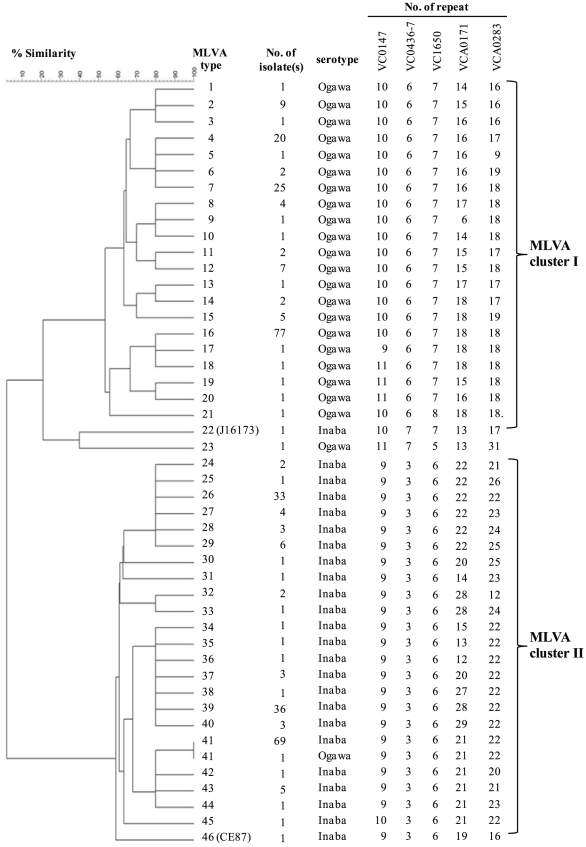
Dendrogram showing genetic similarity between 343 isolates of *V. cholerae* O1 derived from MLVA. Sequence data of repeats on the five loci for each isolate were counted and imported into BioNumerics software version 6.1. Clustering analysis was performed using the unweighted pair group with arithmetic averaging (UPGMA) with a categorical similarity coefficient. *V. cholerae* O1 isolate MLVA types of the El Tor variant were grouped into 2 major clusters, I and II.

MLVA of the 343 isolates resulted in 44 different MLVA types, with a Simpson's index of diversity (SID) of 0.879 (95% CI; range 0.861–0.897) (Figure 2). On the other hand, PFGE differentiated these isolates into 10 PFGE types with a SID of 0.614 (95% CI; range 0.58–0.648). The discriminatory power of MLVA was significantly higher than that of PFGE.

UPGMA clustering of the MLVA profile revealed the existence of two major clusters, I and II (Figure 2). The 164 isolates of serotype Ogawa in PFGE group A sharing a 50% similarity contained 21 MLVA types with a SID of 0.739 (95% CI; range 0.677–0.800), whereas the 177 isolates of serotype Inaba in group B sharing 60% similarity included 22 MLVA types with a SID of 0.772 (95% CI; range 0.729–0.815). MLVA clusters I and II matched up with PFGE groups A and B, and with serotypes Ogawa and Inaba, respectively. It should be noted here that we found one *V. cholerae* O1 carrying the El Tor type *ctxB* which did belong to neither the two known MLVA clusters I and II nor PFGE group A and B.

### Comparison of genotypic variations among isolates from separated outbreak areas over time

**Figure 3:**
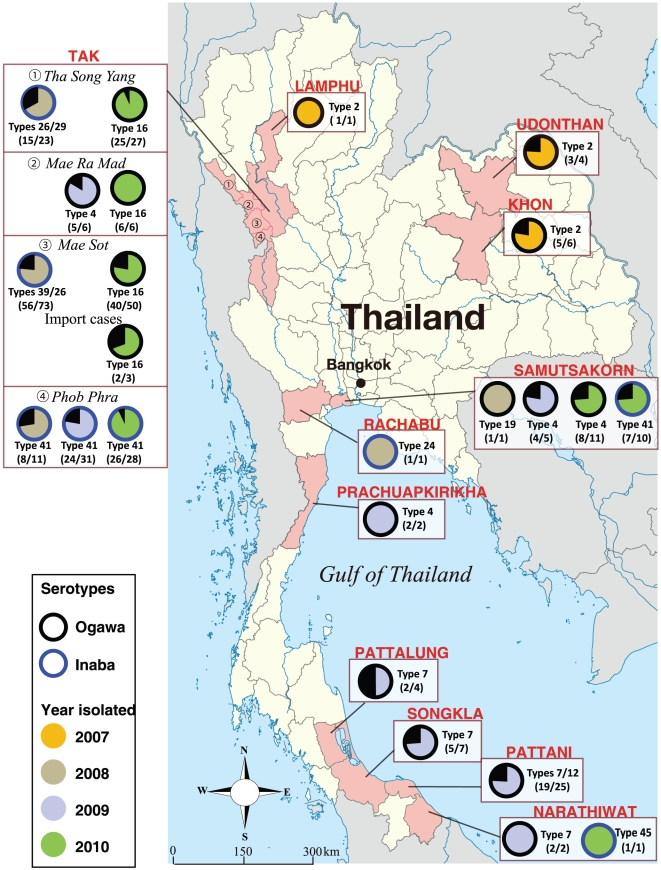
Distribution of major MLVA types of *V. cholerae* O1 isolates during the 2007–2010 cholera outbreaks in Thailand. The distribution percentage of predominant MLVA type (s) is displayed in relatively scaled pie charts. The size of each chart and the number in parentheses indicate the ratio of each major MLVA type (corresponding to Table 3) to the other types. The color of each slice indicates the year of isolation: 2007 (orange), 2008 (gray), 2009 (light blue), and 2010 (green). The color of the circle band denotes serotype: Ogawa (black) and Inaba (blue).

We compared genotypic variations of the isolates obtained from separate outbreak sites over a period of several years (Table 3). In addition, we divided 43 MLVA types of El Tor variant isolates into Group A/Cluster I and Group B/Cluster II. Nine of 11 outbreak isolates in three provinces, Khon Kaen, Udonthani, and Lamphun (Figure 3), were found to be MLVA type 2 in 2007. These outbreaks were caused by the consumption of contaminated cockles. MLVA type 7 was predominant in the 2009 outbreaks of the southern coastal areas, including Patthalung, Songkla, Pattani, and Narathiwat provinces and MLVA type 4 was predominant in the 2009 and 2010 outbreaks of the central coastal areas of Thailand, including Samutsakorn and Prachuapkirikhan provinces. These major MLVA types 2, 7, and 4 were noted to accompany with others as they are closely related, differing by only a single repeat (Table 3).

**Table 3:** MLVA types and pulsotypes of *V. cholerae* O1 isolates in different province of Thailand over time.

			Group A/Cluster I		Group B/Cluster II	
Province	District	Year	MLVA type(No. of isolates)	Pulsotype	MLVA type(No. of isolates)	Pulsotype
Khon Kaen	-	2007	**2(5)** *, *11(1)* †	A1	-‡	-
Udonthani	-	2007	**2(3)**, *1(1)*	A1	-	-
Lamphun	-	2007	**2(1)**	A1	-	-
Samutsakorn	-	2008	**19(1)**	A1	-	-
	-	2009	**4(4)**, *7(1)*	A4	-	-
	-	2010	**4(8)**, *7(1), 13(1), 3(1)*	A1, A4	**41(7)**, *43(2), 24(1)*	B1
Rachaburi	-	2008	-	-	**24(1)**	B2
Prachuapkirikhan	-	2009	**4(2)**	A1	-	-
Songkla	-	2009	**7(5)**, *11(1), 12(1)*	A4	-	-
Pattani	-	2009	**7(14)**, **12 (5)**, *6 (2), 8 (1), 4(1), 9 (1)*, 10 (1)	A1, A4, A5	-	-
Patthalung	-	2009	**7(2)**, *12(1), 20(1)*	A4	-	-
Narathiwat	-	2009	**7(2)**	A4	-	-
	-	2010	-	-	**45 (1)**	B1
Tak	Mae Sot	2008	-	-	**39(35)**, **26(21)**, *27(3), 41(4), 40(3)*, 32(2), *28(1), 38(1), 37(1)*, 29(1), 34(1)	B1
	Tha Song Yang	2008	-	-	**26(10)**, **29(5)**, *28(2), 25(1), 27(1), 41(1)*, 30(1), 39(1), 33(1)	B1
	Phob Phra	2008	-	-	**41(8)**, *37(2)*, 35(1)	B1
	Mae Ra Mad	2009	**4(5)**, *5(1)*	A1, A4	-	-
	Phob Phra	2009	-	-	**41(24)**, *43(3), 44(1), 26(1)*, 42(1), 31(1)	B1
	Mae Sot	2010	**16(40)**, *15(3), 8(2), 14(2), 17(1), 18(1), 21(1)*	A2, A3, A4	-	-
	Mae Sot (Inmport cases)	2010	**16(2)**, *15(1)*	A1, A4	-	-
	Mae Ra Mad	2010	**16(6)**	A1, A4	-	-
	Tha Song Yang	2010	**16(25)**, *8(1), 15(1)*	A1, A2, A3, A4, A5, A6, A7	-	-
	Phob Phra	2010	**16(4)** ¶	A1, A4	**41(26)**, *26(1)*, 36(1)	B1

*Bold type indicates the major MLVA type.

† Italic type represents the MLVA type showing one repeat number difference from all loci of MLVA type shows in bold type.

‡ Not isolated.

¶ These cases are related to a mass food poisoning in a Thai restaurant in the Mae Sot district.

The majority of Group B/Cluster II isolates were from Tak province during 2008 and 2010. We obtained 108 isolates of *V. cholerae* O1 in three districts (Mae Sot, Phob Phra, and Tha Song Yang) of Tak province (Figure 3) during the epidemic period from June through December, 2008. All isolates of the El Tor variant were undistinguishable by PFGE (Table 3) but could be differentiated into 15 types by MLVA. Among the eleven MLVA types in Mae Sot, the majority were types 39 (48%) and 26 (28%); types 38, 40, 34 and 32 were occasionally found in this district. In Tha Song Yang, nine MLVA types were found, including types 26 (43%) and 29 (22%). MLVA type 41 was predominantly found in Phob Phra. Thus, the isolates from the three districts of Tak each exhibited a distinct predominant type.

Although 15 MLVA types of El Tor variant isolates were found in Tak province in 2008, 13 of these disappeared during 2009 and 2010 (Table 3 and Figure S1). While new MLVA types 43 and 44, which differ by only one repeat number from type 41, emerged in Phob Phra during 2009; types 42 and 31 differed by two repeat numbers. In the 2010 large outbreak in Tak, the Group B/Cluster II isolates were completely replaced by the Group A/Cluster I isolates (mainly MLVA type 16), except for the isolates from Phob Phra district. MLVA type 41 was dominant in this district during our surveillance.

## Discussion

This study shows that the cholera outbreaks in Thailand during 2007–2010 were exclusively caused by the *V. cholerae* O1 El Tor variant carrying the classical *ctxB* and El Tor *rstR* genes. PFGE differentiated Thai El Tor variant isolates into nine pulsotypes that share an overall similarity of 88%. UPGMA clustering categorized the PFGE pulsotypes into two major groups, A and B, which is in agreement with the results obtained by MLVA. Arbitrarily selected isolates belonging to group A (A1–A7) were determined to be ribotype III, whereas group B (B1–B2) isolates were of ribotype IV (Table S3). As neither of these two ribotypes were reported in Thailand before [38], [39], the *V. cholerae* O1 variant carrying classical *ctxB* and El Tor *rstR* genes probably appeared in Thailand during recent years.

PFGE pulsotypes and ribotypes of our Thai isolates were also shown to be similar to the predominant types found in India (Table 2, Figure S2). *V. cholerae* O1 ribotype RIII was first identified in 1993, and remained predominant up to 2003 in Kolkata, India [40], [41] from where it spread across the country, eventually reaching Africa [42]–[44]. RIV *V. cholerae* serotype Inaba of pulsotype H1 appeared in India in 2004 and dominated until 2005 [40], [41]. In addition, the presence of a hybrid CTX prophage (classical *ctxB* and El Tor *rstR*) is a unique feature of recent Indian isolates since 1995 [4]. PFGE groups A and B isolates were also found to possess a truncated VSP-II region as seen in reference strains J16173 and CE87 of Indian origin. This feature is unique in a representative El Tor variant strain, CIRS101, which possesses classical *ctxB* and El Tor *rstR* genes; other El Tor variant strains harbor classical *rstR* and *ctxB* genes and complete VSP-II, for example, MJ-1236, a Matlab type I hybrid variant from Bangladesh that cannot be biotyped by conventional methods, and B33, a Mozambique variant that can be biotyped as El Tor [45], [46].

Thai isolate MLVA profiles of 10,6,7,X,X (46.4%, 159/343) and 9,3,6,X,X (51.6%, 177/343; X referring to anonymous repeat number) relate to those of Indian, Bangladeshi and Vietnamese El Tor variant isolates [47], so it is likely that the Thai isolates are derived from a common ancestor of the El Tor variant from India, which eventually spread into Thailand and neighboring countries in recent years.

PFGE and MLVA exhibit a high discrimination power for the differentiation of *V. cholerae* O1 isolates. In this regard, Chun *et al.* [46] reported that 23 *V. cholerae* isolates from different sources over the past 98 years were phylogenetically separated into 12 distinct lineages of which one comprises O1 classical and El Tor bitypes. Their genomics approach concluded that *V. cholerae* undergoes extensive recombination by lateral gene transfer. Other investigators suggested that environmental selection pressure results in a highly heterogeneous population of this bacterial species in a cholera-endemic area, and that few strains appear to evolve into pathogenic clones [48]. In the present study, we obtained 15 isolates of the *V. cholerae* O1 El Tor variant from several environmental sources, such as wells, brooks, drinking/waste water and algae. Notably, MLVA types were closely matched between environmental and human isolates.

One dominant and several minor MLVA types were identified among isolates during outbreak episodes in different geographic regions over time. Most differences between these types were just one repeat number from the tandem repeats of all loci examined (Table 3 and Figure 2). In this regard, Kendall *et al.* [24] examined the *in vitro* genetic relatedness of *V. cholerae* over time. Culturing of three clinical isolates resulted in 18 different lineages with alleles distinct from the original. Seven novel alleles showed an increased number of repeats and 11 had a decreased number of repeats compared with the original, suggesting that the appearance of a dominant MLVA type and closely related MLVA subtypes is likely during an outbreak. Determination of the extent of genetic variation that occurs within multiple circulating MLVA types of *V. cholerae* O1 El Tor can provide a more accurate assessment of outbreak investigations in terms of transmissibility and pathogenic capability.

MLVA typing among isolates revealed geographical and temporal associations of causative *V. cholerae* in cholera outbreaks. The 2007 cholera outbreaks in northeastern Thailand were triggered by the consumption of cockles contaminated with *V. cholerae* O1 MLVA type 2. On the other hand, outbreaks in the southern Gulf areas outbreaks in 2009 were linked mainly with MLVA types 7 and 12, while those in the central Gulf areas during 2009–2010 were linked with MLVA type 4. These MLVA types were very closely related and presumably acquired an additional repeat in the VNTR loci of *V. cholerae* O1 while inhabiting the coastal areas.

In Tak province, more than 80% of the isolates were obtained from Myanmar migrants, with the remainder from local Thai residents, as reported by Swaddiwudhipong *et al.* [49]. Notably, 6 out of 8 isolates from coastal areas were also derived from Myanmar migrants in addition to 2 isolates from Thai residents, and 21 isolates of unknown origin. Although Tak province is far from the coast, the isolates from these areas exhibited the same MLVA types: 4 (in 2009) or 41 (in 2010) (Table 3). The distribution of these MLVA types suggests that cholera epidemics spread quickly by the movement of people across national boundaries. The possibility of *V. cholerae* transmission to other sites by unknown mechanisms should not be eliminated.

We note that MLVA type 41 continued to exist predominantly in an outbreak site of Tak (Phob Phra district) for more than three years (Figure 3, unpublished results). Long-term survival of *V. cholerae* O1 of a particular MVLA type, such as type 41, may be attained in watery environments or in humans who chronically carry the organisms with no signs or symptoms of cholera [50]. Interventions that target critical steps in endemic settings and transmission of causative *V. cholerae* should be taken into consideration for the prevention and control of cholera outbreaks. In this regard, we found a unique *V. cholerae* O1 carrying the El Tor type *ctxB* gene from a Myanmar sick merchant stayed in Tak. PCR analysis showed that the isolate did not possess the VC2346 gene, a specific marker for seventh pandemic clone [32]. Further characterization of this isolate is now in progress in our laboratories.

In summary, we have shown that a combination of PFGE, MLVA and ribotyping provides insights into the genetic background of *V. cholerae* O1 isolates from cholera outbreaks in Thailand during 2007–2010. The results are particularly relevant to the molecular epidemiology studies of *V. cholerae* to trace the emergence, year-long survival, or disappearance of a particular type (s) of isolate in terms of spatial and temporal associations.

## Supporting Information

Figure S1 **Minimum spanning tree of 343 Thai isolates and two reference strains of** ***V. cholerae*** **O1 typed by MLVA.** Each circle in the trees represents a different MLVA type. The size of the circle reflects the number of isolates, and the colors indicate the proportion of isolates obtained during the year. MLVA types 22 and 46 (red circles) are reference strains J16173 and CE87, respectively, of Indian origin. Thick lines represent types differing by a single MLVA locus, thin lines represent 2 types differing by 2 or 3 MLVA loci, dotted lines represent 2 types differing by 4 MLVA loci. Groups A and B of related PFGE types are distinguished.
(EPS)

Figure S2 **Pulsed-field gel electrophoresis of Thai outbreak isolates (pulsotypes A1 and B1) and reference Indian strains (pulsotypes H and H1).**
(EPS)

Table S1 **Characterization of the** ***V. cholerae*** **O1 isolates used in this study.**
(XLS)

Table S2 **PCR primers used in this study.**
(DOC)

Table S3 **Relatedness of** ***V. cholerae*** **O1 El Tor variant isolates classified by several methods.**
(DOC)
