# Supplementary material for: Genotypic and PFGE/MLVA Analyses of Vibrio cholerae O1: Geographical Spread and Temporal Changes during the 2007–2010 Cholera Outbreaks in Thailand
Source: PLoS One. 2012 Jan 24;7(1):e30863. doi: 10.1371/journal.pone.0030863 (PMC3265523; doi:10.1371/journal.pone.0030863)
Supplement: Table S2 — PCR primers used in this study. (DOC) [file pone.0030863.s004.doc]

| **Table S2 PCR primers used in this study** | | | |
| --- | --- | --- | --- |
|  | |  |  |
| **PCR Primer** | | **Nucleotide sequence (5’→3’)** | **Reference** |
| *rtxC* | rtxC-F | CGACGAAGATCATTGACGAC | [28] |
|  | rtxC-R | CATCGTCGTTATGTGGTTGC |  |
| *rstR* | rstR-1F (classical) | CTTCTCATCAGCAAAGCCTCCATC | [29] |
|  | rstR-2F (El Tor) | GCACCATGATTTAAGATGCTC |  |
|  | rstA-3R | TCGAGTTGTAATTCATCAAGAGTG |  |
| *ctxB* | Fw-con | ACTATCTTCAGCATATGCACATGG | [31] |
|  | Rv-cla (classical) | CCTGGTACTTCTACTTGAAACG |  |
|  | Rv-elt (El Tor) | CCTGGTACTTCTACTTGAAACA |  |
| *ctxA* | ctxA-F | CGGGCAGATTCTAGACCTCCTG | [30] |
|  | ctxA-B | CGATGATCTTGGAGCATTCCCAC |  |
| *zot* | zot-F | TCGCTTAACGATGGCGCGTTTT | [30] |
|  | zot-B | AACCCCGTTTCACTTCTACCCA |  |
| *ace* | ace-F | TAAGGATGTGCTTATGATGGACACCC | [30] |
|  | ace-B | CGTGATGAATAAAGATACTCATAGG |  |
| *ompU* | ompU-F | ACGCTGACGGAATCAACCAAAG | [30] |
|  | ompU-B | GCGGAAGTTTGGCTTGAAGTAG |  |
| *toxR* | toxR-F | CCTTCGATCCCCTAAGCAATAC | [30] |
|  | toxR-R | AGGGTTAGCAACGATGCGTAAG |  |
| *tcpA* | tcpA-F | CACGATAAGAAAACCGGTCAAGAG | [30] |
|  | tcpA-B/Clas | TTACCAAATGCAACGCCGAATG |  |
|  | tcpA-B/El | CGAAAGCACCTTCTTTCACACGTTG |  |
| Ch I insertion site | VC0174-F | AAACTGGCGACCTTTGAGCAAGC | [32] |
|  | VC0186-R | GATGGTAGCCTGACGCTGCATCTG |  |
| VC0502 | VC0502F | TCATCAGTTAGCACACGAAC | [33] |
|  | VC0502R | GCTATCGTTATACTTGGCG |  |
| VC0514 | VC0514F | TTATGATCCAAGGAGTAGGG | [33] |
|  | VC0514R | AGGCTGAAAAACAACTTGAG |  |
| VSP2 | pVSP2-IIIF | CCAGCAAACGGTCATTCGCT | [34] |
|  | pVSP2-IIIR | TGGTTGGAAGGTGGGTTGTGT |  |
| VC0147 | VC0147-F | TTGTCATGGCTTGGATTTGG | [22] |
|  | VCO147-R | TGTCGATCACCAATGGCTGC |  |
| VC0436 | VC0436-F | CGTGGTACTAAGTTCCACGC | [22] |
|  | VC0436-R | CGTTTTTACCACGCTCCGCTTC |  |
| VC1650 | VC1650-F | CTACCAAGCGGCGGTTAAGCTG | [22] |
|  | VC1650-R | TGGGCAACCTGCTGGTAGC |  |
| VCA0171 | VCA0171-F | GCATCATCCACAGCGTTTGG | [22] |
|  | VCA0171-R | GCTGAAGCCTTTCGCGATCC |  |
| VCA0283 | VCA0283-F | GTACATTCACAATTTGCTCACCC | [22] |
|  | VCA0283-R | ACTTCAAAACTATTGCGCAC |  |
