# Supplementary material for: Genotypic and PFGE/MLVA Analyses of Vibrio cholerae O1: Geographical Spread and Temporal Changes during the 2007–2010 Cholera Outbreaks in Thailand
Source: PLoS One. 2012 Jan 24;7(1):e30863. doi: 10.1371/journal.pone.0030863 (PMC3265523; doi:10.1371/journal.pone.0030863)
Supplement: Table S3 — Relatedness of V. cholerae O1 El Tor variant isolates classified by several methods. (DOC) [file pone.0030863.s005.doc]

**Table S3 Relatedness of *V. cholerae* El Tor variant isolates classified by several methods**

| **Serotype** | **PFGE** | **MLVA** | **Ribotype^1^** |
| --- | --- | --- | --- |
| **Ogawa** | Group A  (Pulsotypes A1 to A7)  (> 92% similarity) | Cluster I  (21 MLVA types) | RIII |
| **Inaba** | Group B  (Pulsotypes B1 and B2)  (98% similarity) | Cluster II  (22 MLVA types) | RIV |

^1^ Arbitrarily selected isolates from each pulsotype
